# Frequency of Physical Activity-Related Injuries Among Adolescents: An Umbrella Review

**DOI:** 10.3389/phrs.2025.1606767

**Published:** 2025-01-22

**Authors:** Kerttu Toivo, Peter Bakalár, Mari Leppänen, Jari Parkkari, Ensar Abazović, Boštjan Šimunič, Kaja Teraž, Marta Malinowska-Cieślik, Jaroslava Kopčáková, Aurel Zelko, Agnieszka Michalska, Dagmar Sigmundová, Rado Pišot, Armin Paravlić

**Affiliations:** ^1^ Tampere Research Centre of Sports Medicine, UKK Institute, Tampere, Finland; ^2^ Faculty of Sports, University of Presov, Presov, Slovakia; ^3^ Faculty of Sport and Health Sciences, University of Jyväskylä, Jyväskylä, Finland; ^4^ Faculty of Sport and Physical Education, University of Sarajevo, Sarajevo, Bosnia and Herzegovina; ^5^ Institute for Kinesiology Research, Scientific Research Center Koper, Koper, Slovenia; ^6^ Faculty of Sport, University of Ljubljana, Ljubljana, Slovenia; ^7^ Department of Environmental Health, Faculty of Health Sciences, Jagiellonian University, Kraków, Poland; ^8^ Department of Health Psychology and Research Methodology, Faculty of Medicine, University of Pavol Jozef Šafárik, Kosice, Slovakia; ^9^ Faculty of Sports, University of Ljubljana, Ljubljana, Slovenia; ^10^ Department of Community and Occupational Medicine, University Medical Center Groningen, University of Groningen, Groningen, Netherlands; ^11^ Department of Biomedical Foundations of Development and Sexology, Faculty of Education, University of Warsaw, Warsaw, Poland; ^12^ Faculty of Physical Culture, Palacký University, Olomouc, Czechia; ^13^ Faculty of Sports Studies, Masaryk University, Brno, Czechia

**Keywords:** physical activity-related injuries, sports injuries, adolescence, frequency, prevalence, incidence

## Abstract

**Objectives:**

The aim of this umbrella review was to investigate the frequency of physical activity-related injuries (PARI) among adolescents. Our secondary objective was to describe the available reviews of injuries in three settings: organized sports, leisure time physical activity (PA), and school physical education (PE).

**Methods:**

We conducted an overview of reviews consistent with the Preferred Reporting Items for Systematic Reviews and Meta-Analyses (PRISMA) statement guidelines on the frequency of physical activity related injuries in adolescents.

**Results:**

We identified 19 systematic reviews with at least moderate quality to include in the review. We were not able to pool data from reviews and conduct meta-analysis due to heterogeneity of injury definitions, exposure times, and sample demographics. All reviews included studies of injuries sustained in organized sports, and injury incidence was higher during matches than training. No systematic reviews were found focusing on leisure time or school-based PA injuries.

**Conclusion:**

High-quality research is essential to understand the frequency of various types of physical activity related injuries among adolescents in organized sports, PE, and leisure time activities to develop more effective prevention strategies.

## Introduction

Regular physical activity (PA) is one of the most cited factors for the prevention of major noncommunicable diseases [1]. During adolescence, PA contributes to health and development of the musculoskeletal system, cardiovascular system, and maintenance of optimal body mass [2–4]. Regular PA is also associated with numerous psychological and social benefits [2, 5, 6].

It is worth noting that PA as a health promotion tool is not one without potential adverse effects [7, 8]. There are numerous epidemiological studies that describe the incidence and burden of PA-related injuries sustained during high-intensity sports and also during recommended types of lower intensity PA [8–10]. Even though deaths caused by participation in PA are uncommon, there are other serious and long-term health consequences of physical activity related injuries, such as injuries to the spine, joints, and traumatic brain injuries [11–13]. At the same time, these injuries have direct costs from evaluation, treatment, and rehabilitation and indirect costs with lost productivity if the injury results in the adolescent missing school or the parent missing work [14]. Furthermore in addition to these, the injured individuals may experience negative psychological and daily living consequences such as frustration, study issues, and sleep disturbances, as well as a loss of identity [15]. Injuries may also cause drop-out from sports and additionally contribute to the decrease in PA levels in the transition from adolescence to adulthood [16, 17]. It also emphasizes the importance of injury prevention that a previous injury increases the risk of experiencing a new injury [10, 18].

Active injury prevention and recognition of injury risk factors needs to be an integral component of PA promotion [8]. This aligns with EU Physical Activity Guidelines (2008) which state that while implementing interventions or programmes designed to increase PA there is a need for evaluation and risk assessment of “overall balance between benefits and possible increased risks of higher levels of PA (e.g., injuries).” The primary aim of this overview of reviews was to systematically map the extent of the physical activity related injury problem in the adolescent population by investigating the incidence and prevalence of physical activity related injuries. Our secondary objective was to describe the available reviews of injury incidence and prevalence in three settings where physical activity related injuries occur: organized sports (high-school and college sports, sports clubs from recreational to elite level, sports academies), leisure-time PA (non-organized sports), and school PE. By doing so, the review aims to provide decision-makers with data overview which can be used in the process of setting the policy actions within physical activity related injury prevention in adolescents.

## Methods

### Search Strategy

This systematic review was undertaken in accordance with the Preferred Reporting Items for Systematic Reviews and Meta-Analyses (PRISMA) statement guidelines [19]. Thus, a systematic search of the research literature published in peer-reviewed journals was conducted for systematic review articles with or without meta-analysis studying the epidemiology of physical activity related injuries among children and adolescents in different settings. To carry out this review, English language literature searches of PubMed, CINAHL, Web of Science, Embase, SPORTDiscus, PEDro and Cochrane Register of Controlled Trials were conducted from April 2021 up to June 2021. Electronic databases were searched using the following keywords and their combinations: “sport injury” “athletic injury,” “physical activity,” “Injury occurrence,” “Injury prevalence,” “epidemiology,” “leisure time activity,” “Children,” “school,” “youth,” “Adolescents,” “Review,” “Meta-analysis.” The reference lists of each included article were also scanned to identify additional relevant studies. The study protocol was prospectively registered at PROSPERO registry: CRD42021247312.

### Inclusion and Exclusion Criteria

The target population of the selected review articles was adolescents from 10 to 19 years of age (WHO definition), with no gender restriction, studies where age groups were overlapping and if it was not possible to extract information of only adolescents the studies were excluded. The outcomes were injury occurence/incidence and injury prevalence. Studies were excluded according to the following criteria: (a) studies written in languages other than English; (b) original studies (c) studies that included other populations than adolescents and it was not possible to extract information of only adolescents; (d) studies from which we could not extract values of interest like RRs, IRs, % of injuries or number of people who got injured. The risk of bias in systematic reviews and assessment of multiple systematic reviews (AMSTAR 2) tool was used to evaluate risk of bias and quality of included articles [20]. We adjusted the AMSTAR 2 tool to be suitable for assessing the quality of descriptive studies or analytical observational studies of health outcomes. From questions 1 and 8 we removed the components of PICOS [21] (intervention and comparator group) that are not applicable to descriptive or observational studies. In questions 3, 11, 12, and 13 we removed randomized controlled trials and non-randomized studies from the answer options. Question 9 was rephrased as “Was a suitable tool used to evaluate the quality of included studies? (Supplementary Material S1). Only studies with moderate to high quality score (≥6.5), were included for the purpose of the present study.

### Screening Strategy

**FIGURE 1:**
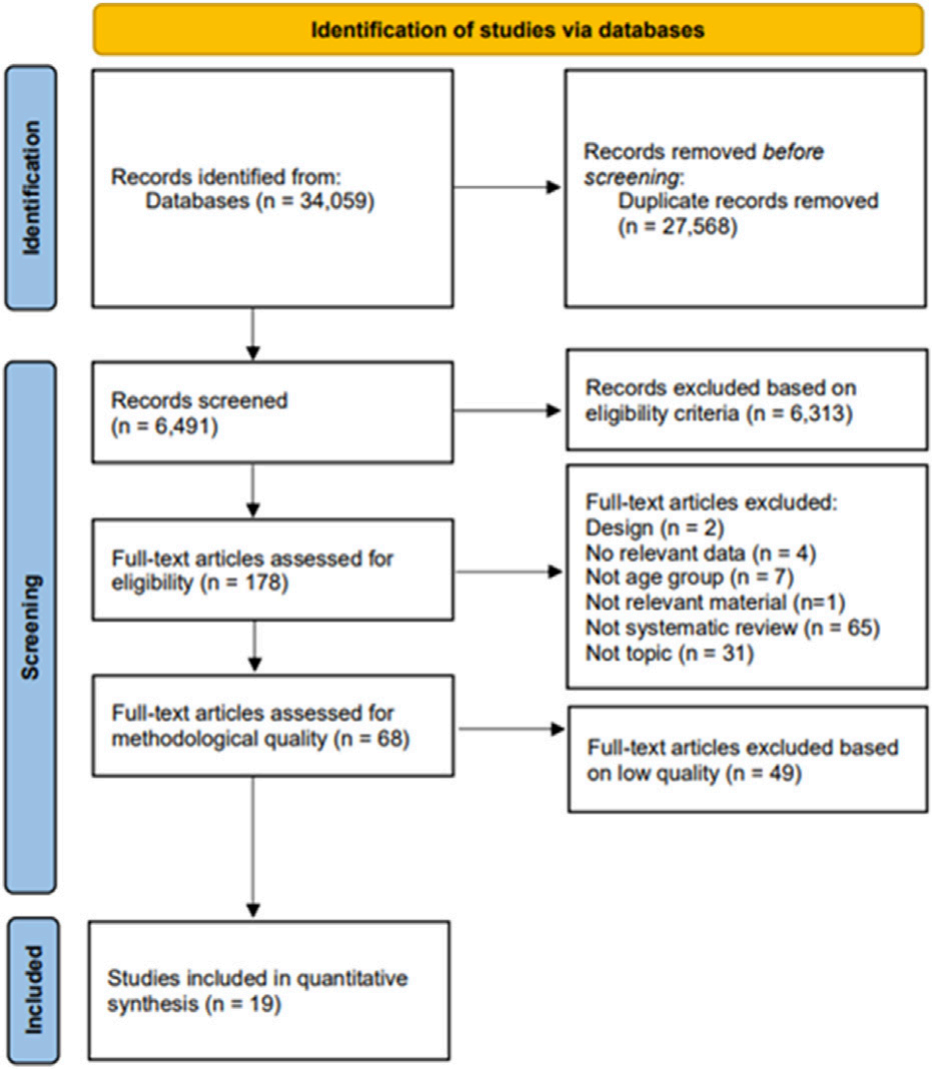
Preferred Reporting Items for Systematic Reviews and Meta-analysis flow-chart. Slovakia, Finland, Slovenia, Poland, Czechia, 2021–2023.

Six independent reviewers (DS, PB, BS, EA, ML, KT) performed the literature search, identification, screening and data extraction. First, the titles were initially screened by the reviewers during the electronic searches to assess the papers’ suitability, and all papers beyond the scope of this review were excluded. Second, the abstracts were assessed using predetermined inclusion and exclusion criteria. Third, the full texts of the remaining papers that met the inclusion criteria were retrieved and included in the ongoing procedure and reviewed by the two reviewers (PB and KT) to reach a final decision on inclusion in the review. Finally, the reference lists from the retrieved manuscripts were also examined for any other potentially eligible papers. Any disagreements between the reviewers were resolved by consensus or arbitration through a third reviewer (AP). If the full text of any paper was not available, the corresponding author was contacted by mail or ResearchGate. The study selection process as described above is illustrated in Figure 1.

### Data Extraction

The Cochrane Consumers and Communication Review Group’s data extraction protocol was used to extract the participant information, including sex, age, sample size, training status, description of the intervention, study design and study outcomes [22]. This was undertaken by one author (AP), while a second author (EA) checked the extracted data for accuracy and completeness. Disagreements were resolved by consensus or by a third reviewer (PB). Reviewers were not blinded to authors, institutions, or manuscript journals.

## Results

### Study Selection

A total of 34,059 articles were identified by the literature search (Figure 1). Following the removal of duplicates and the elimination of articles based on title and abstract screening, 178 studies remained. An evaluation of the remaining 178 studies was conducted independently by two researchers (PB and KT). The list of excluded studies after full texts assessed for eligibility, with reasons for exclusion, can be found in Supplementary Material S2. The list of excluded studies after quality assessment can be found in Supplementary Material S3. Following the final screening process, 19 studies were included in the review and these were published in the year 2014 or later.

### Methodological Quality of Included Studies

**FIGURE 2:**
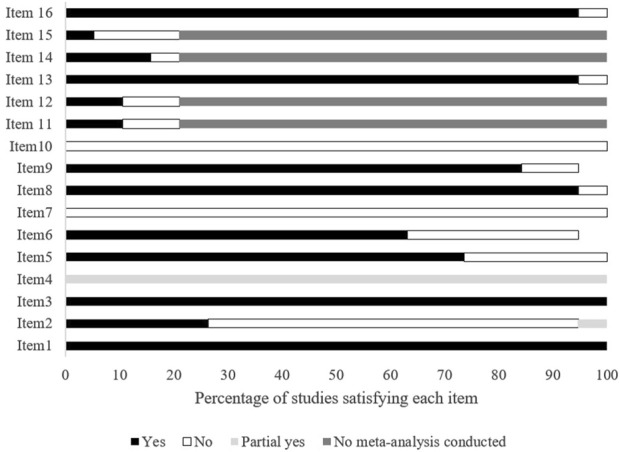
Overall results of the modified version of Assessing the Methodological Quality of Systematic Reviews. Slovakia, Finland, Slovenia, Poland, Czechia, 2021–2023.

The methodological quality of the included reviews is depicted in Figure 2. On average, the included studies were of moderate quality, with an AMSTAR-2 score of 8.3, ranging from 6.5 to 10.5. All studies described their research question and inclusion criteria using relevant PICO components (Item 1) and explained the desired study designs for inclusion in the review (Item 3). Additionally, all studies partially met the criteria for using a comprehensive search strategy (Item 4). However, none of the reviews provided a list of excluded studies to justify the exclusion reasons (Item 7) or reported the sources of funding for the included studies (Item 10).

### Study Characteristics

All selected studies were designed as systematic literature reviews, and meta-analysis was conducted in two articles to summarize the findings [23, 24]. In total, 572 original studies were included in the reviews. The majority of reviews investigated injury incidence and prevalence in both sexes, whereas only two studies aimed to investigate this question specifically for females [25] or males [26]. Injuries sustained in a single sport were investigated in the majority of reviews, these sports were rugby [25, 27], baseball [28], artistic gymnastics [29], cricket [30, 31], football [26], handball [32], fieldhockey [33], netball [34], and swimming [35]. Seven reviews included a variety of sports, such as baseball and softball [36], action sports (snowboarding, alpine skiing, motorsports etc) [24], or several different types of sports [23, 37–40]. Nine studies investigated epidemiology of a variety injury types within a selected sport [25, 26, 29–34, 41], while others focused on particular types of injuries such as concussion or traumatic brain injury [24, 27, 36, 39], ankle sprain [23], elbow disorders [28], back pain [40], shoulder pain [35], and overuse injuries of the extremities [38] or overuse injuries of wrist alone [37]. Five reviews included only studies with prospective injury surveillance [23, 26, 28, 30, 33].

### Results on Injury Incidence and Injury Prevalence

Thirteen articles reported data on injury incidence, whereas six articles reported data on injury prevalence (Table 1). The large heterogeneity on reporting the injury incidence was observed, thus making it difficult to quantitatively summarize data on injury incidence. Five studies presented data on incidence per 1,000 player-hours [27, 32–34, 41], 1,000 days of real exposure [24], per 1,000 exposures [23, 28], per 1,000 h of playing/match/training exposure [25, 26], per 100.000 people [39], per athlete [29], or athlete exposure [38]. The highest incidence of 90.9 per 1,000 player-hours was reported in field hockey during the African Cup of Nations [33], whereas lowest overall injury incidence was reported in handball ranging from 1.7 to 7.8 per 1,000 player-hours [32]. Five studies [25, 32, 38, 39, 41] examined differences in injury incidence during match and training settings. Raya-Gonzalez and colleagues [32] investigated injury incidence in handball and found that more injuries are reported during match than training (match: 10.8 to 73.6 vs training: 0.96 to 4.1 per 1,000 player-hours). The similar pattern was observed in rugby [41], where 3.7 to 108.5 vs 0.4 to 56.2 injuries occurs per player-hours during match and training, respectively. Four studies presented data on injury incidence for different age categories [23, 28, 32, 33], while three studies examined the influence of the athletes’ sex on the risk of injury [23, 29, 32]. In handball, senior female and youth male athletes reported greater injury incidence compared to youth female handball players (13.0–36.0 vs. 10.8–23.8), but higher compared to senior male handball players (13.0–36.0 vs 15.0 to 73.6 per player-hours) [32]. On the contrary, Doherty and colleagues [23] reported higher injury incidence in females than males (13.6 vs. 6.94 per 1,000 exposures), with also higher injury incidence in children than adolescents (2.85 vs. 1.94 per 1,000 exposures), and higher injury incidence in adolescents than adult athletes (1.94 vs. 0.72 per 1,000 exposures) respectively.

**TABLE 1:** Included review articles with study characteristics and results. Slovakia, Finland, Slovenia, Poland, Czechia, 2021–2023.

Reference	Country	Study type	No of included studies	Setting	Age	Sex	Type of injuries	Outcome	Prevalence	Incidence	IRR	Reporting statistics
2014 Doherty The Incidence and Prevalence of Ankle Sprain Injury - A Systematic Review and Meta-Analysis of Prospective Epidemiological Studies	Ireland	SLR with MA	181	Variety of sports	all	both	Ankle sprain	Incidence and prevalence	Pooled prevalence (perdiod varied between studies) a) indoor/court sports 12.17% b) water/ice sports 4.36% c) field-based sports 11.3% d) outdoor pursuits sports 11.65%	a) Females vs Males (13.6 vs. 6.94 per 1,000 exposures) b) children vs Adolescents (2.85 vs. 1.94 per 1,000 exposures) c) adolescents vs adults (1.94 vs. 0.72 per 1,000 exposures)		per 1,000 exposures
2015 Kirkwood Concussion in youth rugby union and rugby league: a systematic review	UK	SLR	25	Rugby	Children and adolescents	both	Concussion	Incidence		INCIDENCE: range between: a) 0.2 to 6.9 per 1,000 player-hours for rugby union b) 4.6 and 14.7 concussions per 1,000 player-hours for rugby league		per 1,000 player-hours
2015 Kox Prevalence, incidence and risk factors for overuse injuries of the wrist in young athletes: a systematic review	Netherlands	SLR	12	Variety of sports	Children and adolescents	both	Overuse injuries - wrist only	Incidence and prevalence	PREVALENCE: a) 32/period not specified)–73% (within 6 months) for wrist pain; b) 10 (current chronic injury)–28% (within 10 years previous to data collection) for overuse wrist injury	INCIDENCE RATES: a) 7%–9% for wrist pain; b) 0.02%–26% for overuse wrist injury		
2016 Chéron Association between sports type and overuse injuries of extremities in children and adolescents: a systematic review	France	SLR	9	Variety of sports	Children and adolescents	both	Overuse injuries	Incidence		INCIDENCE: range between: a) 0.3 to 0.5 PROPORTIONS 0.04 to 1.8 per 1,000 h of exposure	Injury rate ratio: a) Total - 0.03 to 1; b) Games - 0.04 to 0.42 per athletic exposure c) Practice - 0.01 to 0.18 to AE	per athlete exposure
2017 Cusimano Systematic Review of Traumatic Brain Injuries in Baseball and Softball: A Framework for Prevention	Australia	SLR	29	Baseball and softball	all	both	Concussion					
2017 Trompeter Prevalence of Back Pain in Sports: A Systematic Review of the Literature	Germany	SLR	43	Variety of sports	all	both	Low back pain	Prevalence	PREVALENCE: a) lifetime prevalence 1%–94%; highest prevalence in rowing and cross-country skiing b) point prevalence 18%–65%; lowest prevalence in basketball and highest prevalence in rowing			
2018 Barboza Injuries in Field Hockey Players: A Systematic Review	Netherlands	SLR	22	Hockey	all	both	all	Incidence		a) school players 0.01 per 1,000 player-hours b) Africa Cup of Nations 90.9 per 1,000 player-hours c) high school women 1 per 1,000 player-hours d) under 21 age women 70 per 1,000 player-hours		per 1,000 player-hours
2019 Campbell Injury epidemiology and risk factors in competitive artistic gymnasts: a systematic review	Australia	SLR	22	Artistic gymnastics	all	both	all	Prevalence	PREVALENCE: a) all - 2.0 to 2.3 per gymnast; b) female - 2.0 (injuries per gymnast year or 1,000 exposure hours) to 2.3 (over 4 years); male - 2.0 (per gymanst year)	INCIDENCE:a) all - 0.3 to 3.6 per gymnast; b) female - 0.3 to 3.6; male - 0.7		per athlete
2019 Jones Injury Incidence, Prevalence and Severity in High‐Level Male Youth Football: A Systematic Review	UK	SLR	23	Football	8–21 years old	male	all	Probability of sustaining an injury and incidence per 1,000 h of exposure		INCIDENCE per 1,000 h of exposure match + training a) youth players aged under 9 to under 21 - 5.8 b) older (under 17–under 21 years) - 7.9 c) younger aged players (under 9–under 16) - 3.7 16 years) TRAINING IRR: a) all age groups 0.69 to 7.9 per 1,000 h; MATCH IRR −0.4 to 80.0 per 1,000 h		per 1,000 h of playing/match/training exposure
2019 King Match and Training Injuries in Women’s Rugby Union: A Systematic Review of Published Studies	Australia	SLR	10	Rugby	14–43	female	all	Incidence		INCIDENCE Rugby 15s - MATCH a) 19.6 per (17.7 TO 21.7) 1,000 match-hours; PLAYING HRS - TRAINING + MATCH b) 3.6 to 37.5; Rugby 7s - MATCH a) 62.5 per (54.7 TO 70.4) 1,000 match-hours; PLAYING HRS - TRAINING + MATCH b) 46.3 to 95.4		per 1,000 h of playing/match/training exposure
2020 Feijen Swim-Training Volume and Shoulder Pain Across the LifeSpan of the Competitive Swimmer: A Systematic Review	Belgium	SLR	12	Swimming	all	both	shoulder pain	Prevalence	PREVALENCE: a) young - 20.0% (point prevalence and within past week); b) adolescents - 91.3% (past month) c) adult - 70.3% (point prevalence and within past week) d) masters - 19.4% (point prevalence and within past week)			
2020 Feletti The Incidence of Pediatric and Adolescent Concussion in Action Sports: A Systematic Review and Meta-Analysis	Italy	SLR with MA	19	Action sports (snowboarding, motocross, alpine skiing etc)	0–19	both	Concussion	Incidence		INCIDENCE 0.0194 to 39.2 per 1,000 days of real exposure		per 1,000 days of real exposure
2020 Grant Incidence and risk factors for musculoskeletal disorders of the elbow in baseball pitchers: a systematic review of the literature	Canada	SLR	39	Baseball	all	male	elbow	Incidence		INCIDENCE: a) youth pitchers - 40.6%; 2.2 per 1,000 exposures b) adolescent pitchers - 2.3% c) professional - 13.5%–21%		per 1,000 exposures
2020 McLeod Medical-attention injuries in community cricket: a systematic review	Australia	SLR	6	Cricket	all	both	all	Incidence and prevalence				
2020 McLeod Prospective reporting of injury in community-level cricket A systematic review to identify research priorities	Australia	SLR	13	Cricket	all	both	all	Injury rate, Incidence and prevalence	PREVALENCE 21 (over 3 consecutive seasons, age U14)-44.7% (lumbar spine injuries over one season, mean age 14.9 years)			
2020 Raya-González Injury Profile of Male and Female Senior and Youth Handball Players: A Systematic Review	Spain	SLR	15	Handball	all	both	all	Incidence		INCIDENCE: a) overall - 1.7 to 7.8 per 1,000 h of exposure; b) MATCH - senior male - 15 to 73.6 - senior female −13 to 36 - youth male - 15.9 to 32.7 - youth female - 10.8 to 23.8; c) TRAINING: overall - 0.96 to 4.1 per 1,000 h of exposure		per 1,000 player-hours
2020 Theadom Incidence of Sports-Related Traumatic Brain Injury of All Severities: A Systematic Review	New Zealand	SLR	11	Variety of sports	all	both	Concussion	Incidence		INCIDENCE 3.5 to 170 per 100,000 people		per 100,000 people
2021 Downs Injuries in Netball-A Systematic Review	Australia	SLR	46	Netball	all	both	all	Injury rate, incidence		INCIDENCE: a) all injuries - 11.3 to 14 per 1,000 player hours; b) ankle sprains - 1.74 c) lower limb injuries - 11.9 d) ankle and knee injuries - 5.9		a) injury incidence per 1,000 player hours b) studies reported injury rates per player, per 1,000 players, per player/match or per player/season
2015 Freitag Systematic review of rugby injuries in children and adolescents under 21 years	UK	SLR	35	Rugby	Children and adolescents	both	all	Incidence		INCIDENCE: a) overall - 26.7 per 1,000 player-hours; b) MATCH 3.7 to 108.5 per 1,000 player-hours; c) TRAINING 0.4 to 56.2 per 1,000 player-hours		per 1,000 player-hours

Finally, injury prevalence was reported for different sports including cricket, swimming, artistic gymnastics, and a variety of sports together. Also, prevalence of different types of injuries was examined such as overuse injuries of wrist only [37], low back pain [40], shoulder pain [35] or all injury related complaints.

Trompeter and colleagues investigated lower back pain prevalence in different sports and reported highest lifetime prevalence in rowing and cross-country skiing (1%–94%) [40]. A study by Kox and colleagues revealed a prevalence ranging from 32 (period not specified) to 73% (within 6 months previous to survey) for wrist pain and 10 (current) to 28% (within 10 years) for wrist overuse injury, respectively [37]. Feijen and colleagues explored the prevalence of shoulder pain in swimmers belonging to different age groups and found the highest prevalence of shoulder pain in adolescent swimmers (91.3% within the past month), followed by adults (70.3%), young (20.0%), and masters (19.4%) combined from within the past week and point prevalence [35].

### Settings of Physical Activity Related Injuries

The majority of reviews encompassed studies of organized sports conducted in sports club environments as well as in school sports settings [24, 25, 27, 30, 31, 35–37, 41]. Some also included professional sports [28, 33, 40]. The school sports were mainly from high school or college settings [24, 25, 35, 36].Three reviews included studies conducted in an opera school [37], vocational dance school [38], and a football academy environment [26]. Studies where gymnasts were recruited from school teams were excluded from the review of injuries in competitive artistic gymnasts [29]. One review focused solely on injuries at elite and national level in handball [32], one focused on injuries in high level male youth football [26], while the reviews by McLeod et al were confined to community cricket excluding high performance centers [30, 31].

None of the reviews were limited solely to leisure time physical activity injuries, although studies that were based on emergency room, medical clinic, or hospital records also included injuries from leisure time activities [24, 34, 36, 39, 41]. Some studies also included studies based on insurance claims or national electronic injury surveillance systems [24]. None of the reviews encompassed only studies of injuries sustained in school PE.

## Discussion

Out of the 19 reviews included, nine reviews focused on a single sport and seven reviews encompassed a variety of sports. Twelve reviews included a variety of injury types and seven focused on injuries of certain type or location. We were not able to pool data and conduct meta-analysis of injury epidemiology due to heterogeneity across studies in injury definition, sample demographics, and exposure times.

A key cause of heterogeneity in epidemiologic physical activity related injury studies is the variety of injury definitions used across studies [42]. A consensus statement by the International Olympic Committee was published in 2020 to strengthen consistency in data collection, injury definitions, and research reporting [43]. It was anticipated that sport and setting specific statements would later be published, and this was published for football in 2022 to ensure more consistent study designs, data collection procedures and use of nomenclature [44]. It should be acknowledged that reporting reduced ability to participate in full training or competition yields a significantly higher incidence of injuries than methods based on time-loss or medical attention and gives a superior view of the scope of the problem for the purpose of injury prevention and monitoring its effectiveness [45]. Further, the wording in an injury questionnaire should also be considered. For example, using the phrase “wrist pain” instead of “overuse injury of the wrist” reveals a higher prevalence [37]. It is also advised to consider that some injuries present as sudden onset but have a repetitive underlying mechanism and methods to capture these should be developed and implemented [43].

To facilitate the comparison of research results, sports injury incidence should be reported as the number of sports injuries per exposure time [46]. Exposure time is frequently reported as athlete exposures, meaning one athlete participating in one sporting session or exposure hours, meaning one athlete being exposed to sustaining a sporting injury for 1 hour. The player hours should be recorded separately for matches and for training, and injury incidence is commonly higher during matches or games than training [25, 32, 38, 41]. The type of sport also has an impact on how the exposure times are frequently reported. In the review of concussions occurring in action sports, such as alpine sports and equestrian sports, exposure times were typically reported as days of real exposure [24] as opposed to hours. The exposure times in some leisure time sports are more difficult to report as these sports are infrequently practiced on a regular basis, for example, daily skiing for 1 week during vacation. The heterogeneity of exposure times in the reviews prevented us from pooling results and conducting meta-analysis.

The design used for injury surveillance (prospective or retrospective) influences the reliability of data collection [46]. With retrospective data collection longer recall periods underestimate injury rates. For more severe injuries, a recall period of 12 months doesn’t affect rate estimates, but for less severe injuries a recall period of no longer than 3 months is recommended [47]. Only five out of the 19 reviews exclusively included studies with a prospective design. These included a review of high-level male youth football injuries in an academy environment [26], a review of injuries in community-level cricket [30], a review of injuries sustained in field hockey reporting the highest injury incidence [33], a review and meta-analysis of ankle sprain injuries in a variety of sports comparing age-groups and sex differences [23], and a review of elbow injuries in baseball pitchers [28]. Prospective study designs should be used in different types of settings whenever possible to minimize the risk of bias.

A significant strength of this review was considering the different settings of physical activity in adolescents considering differences between countries and the identification of research gaps in this area. In some countries both recreational and competitive sports for adolescents are primarily organized in schools, whereas in other countries sports clubs are the most common way to participate in organized sports [48], and school related PA is limited to PE classes. Further, in some countries adolescents rotate from one sport to another during the school year, whereas it seems to be increasingly common to specialize at an early age and participate in the same sport all year round, which may increase the injury risk [49]. In this review we classified high-school and college sports as organized sports as they are different from school PE classes. There are few retrospective questionnaire studies of adolescents physical activity related injury epidemiology comparing different settings in which the injury has occurred, namely, organized sports, school PE, and leisure time activities, with the recall period ranging from 1 week to 1 year [7, 50, 51]. We did not find any reviews of only leisure time physical activity related injuries or PE related injuries in adolescents. Thus, prospective studies investigating this issue are warranted.

All the 19 reviews of at least moderate quality reviews included in this study were published in the year 2014 or later. As tools for evaluating the quality of reviews emerge, these are also used to guide writing of reviews in times ahead. The first AMSTAR criteria were published in 2007 and AMSTAR-2 10 years later [20]. Potentially some information from high quality reviews conducted at an earlier point in time may be excluded, as we did not evaluate the quality of individual studies included in the reviews. This represents a potential limitation of the present umbrella review. On the other hand, it is important to collect current information on injury incidence to reveal the effect of injury preventive methods-such as rule changes and obligatory use of protective wear [52, 53]. Another limitation is the relatively small number of articles included in the review which may limit the generalizability of the results.

Besides for the purpose of planning effective measures to prevent injuries and monitoring their effect [54], epidemiologic studies of PARI can be utilized for organizing medical services in different types of healthcare settings especially in the proximity of sporting environments such as at a football tournament or ski resort. The injury prevalence is higher in adolescents participating in organized sports compared to non-participants [55] and neuromuscular training (NMT) being effective in the prevention of injuries should be included as part of the normal training in organized adolescents sports [56]. NMT has also been shown to be effective in a school setting [57]. Further, especially in school PE, the risk of injury is increased among inactive adolescents [50]. In addition to NMT; other effective measures for sports injury prevention include rule modification and equipment recommendations [49, 58]. The responsibility for prevention of PARIs in adolescents is divided between organized sports, schools, families, as well as decision makers [59].

The basis for a physically active lifestyle is formed during childhood and adolescence and PARIs may temporarily or permanently weaken the individuals’ ability to be able to enjoy sports participation or other leisure time physical activities [60]. Physical activity related injuries can have long-term negative effects on athletes’ development, leading to early cessation of their athletic careers and the development of first signs of depression and other mental health problems. To better understand the scale of the problem related to PARI, large-scale injury surveillance studies with similar methodologies need to be conducted in different settings. These studies are necessary to provide current and reliable data on physical activity related injuries in adolescents [61].
