# Supplementary material for: Frequency of Physical Activity-Related Injuries Among Adolescents: An Umbrella Review
Source: Public Health Rev. 2025 Jan 22;46:1606767. doi: 10.3389/phrs.2025.1606767 (PMC11815350; doi:10.3389/phrs.2025.1606767)
Supplement: Supplementary file 2 [file Table2.docx]

Supplementary file 2: List of excluded studies after full-texts assessed for eligibility (n = 111)

| **Study** | **Title** | **Reason for exclusion** |
| --- | --- | --- |
| Kvist et al., 1989 | Sports-Related Injuries in Children | Not systematic review |
| Runyan and Gerken, 1989 | Epidemiology and Prevention of Adolescent Injury. A Review and Research Agenda | Not systematic review |
| Taimela et al., 1990 | Intrinsic Risk Factors and Athletic Injuries | Not systematic review |
| Van Mechelen et al., 1992 | Incidence, Severity, Aetiology and Prevention of Sports Injuries. A Review of Concepts | Not systematic review |
| Bijur et al., 1995 | Sports and Recreation Injuries in US Children and Adolescents | Not systematic review |
| Pasque et al., 2000 | A Prospective Study of High School Wrestling Injuries | Not systematic review |
| Patel and Nelson, 2000 | Sports Injuries in Adolescents | Not systematic review |
| Belechri et al., 2001 | Sports Injuries Among Children in Six European Union Countries | Not systematic review |
| Burt and Overpeck, 2001 | Emergency Visits for Sports-Related Injuries | Not systematic review |
| Söderman et al., 2001 | Injuries in Adolescent Female Players in European Football: A Prospective Study Over One Outdoor Soccer Season | Not systematic review |
| Kirialanis et al., 2002 | Injuries in Artistic Gymnastic Elite Adolescent Male and Female Athletes | Not systematic review |
| Damore et al., 2003 | Patterns in Childhood Sports Injury | Not systematic review |
| Emery, 2003 | Risk Factors for Injury in Child and Adolescent Sport: A Systematic Review of the Literature | Not topic |
| Skokan, et al. 2003 | Serious Winter Sport Injuries in Children and Adolescents Requiring Hospitalization | Not systematic review |
| Lord and Winell, 2004 | Overuse Injuries in Pediatric Athletes | Not systematic review |
| Hinton et al., 2005 | Epidemiology of Lacrosse Injuries in High School–Aged Girls and Boys. A 3-Year Prospective Study | Not systematic review |
| Sundblad et al., 2005 | Injuries During Physical Activity in School Children | Not systematic review |
| Ramirez et al., 2006 | Injuries to High School Football Athletes in California | Not systematic review |
| Fernandez et al., 2007 | Epidemiology of Lower Extremity Injuries among U.S. High School Athletes | Not systematic review |
| Visser et al., 2007 | Accident Proneness, Does it Exist? A Review and Meta-analysis | Not topic |
| Kemp et al., 2008 | Patterns of Skeletal Fractures in Child Abuse: Systematic  Review | Not topic |
| Schanmugan and Maffulli, 2008 | Sports Injuries in Children | Not systematic review |
| Faigenbaum et al., 2009 | Youth Resistance Training: Updated Poisiton Statement Paper From the National Strenght and Conditioning Association | Not systematic review |
| Frisch et al., 2009 | Injuries, Risk Factors and Prevention Initiatives in Youth Sport | Not systematic review |
| Lystad et al., 2009 | Epidemiology of Injuries in Competition Taekwondo - A Meta-analysis of Observational Studies | No relevant data |
| McHugh, 2009 | Oversized Young Athletes: A Weight Concern | Not systematic review |
| Thompson et al., 2009 | Helmets for Preventing Head and Facial Injuries in Bicyclist (Review) | Not topic |
| Cuff et al., 2010 | Overuse Injuries in High School Athletes | Not systematic review |
| Haseler et al., 2010 | The Epidemiology of Injuries in English Youth Community Rugby Union | Not systematic review |
| Janssen and LeBlanc, 2010 | Systematic Review of the Health Benefits of Physical Activity and Fitness in School-aged Children and Youth | Not topic |
| MacPherson and Spinks, 2010 | Bicycle Helmet Legislation for the Uptake of Helmet Use and Prevention of Head Injuries | Not topic |
| Maffulli et al., 2010 | Sport Injuries: A Review of Outcomes | Not systematic review |
| Monroe et al., 2011 | Most Common Sports-Related Injuries in a Pediatric Emergency Department | Not systematic review |
| Wood et al., 2010 | The Epidemiology of Sports-related Fractures in Adolescetns | Not systematic review |
| Brito et al., 2011 | Injuries in Youth Soccer During the Preseason | Not systematic review |
| Caine and Golightly, 2011 | Osteoarthritis as an Outcome of Paediatric Sport: An Epidemiological Perspective | Not systematic review |
| Gottschalk et al., 2011 | Epidemiology of Sports Injury in Pediatric Athletes | Not systematic review |
| Shanley et al., 2011 | Incidence of Injuries in High School Softball and Baseball Players | Not systematic review |
| Tenforde et al., 2011 | Overuse Injuries in High School Runners: Lifetime Prevalence and Prevention Strategies | Not systematic review |
| Habelt et al., 2011 | Sport Injuries in Adolescents | Not systematic review |
| Edouard et al., 2012 | Injuries in Youth and National Combined Events Championships | Not systematic review |
| Guerriero et al., 2012 | Epidemiology, Trends, Assessment and Management of Sport-related Concussion in United States High Schools | Not systematic review |
| Kraus et al., 2012 | The Epidemilogy of Knee Injuries in Children and Adolescents | Not systematic review |
| Ruedl, et al. 2012 | Sport Injuries and Illnesses During the First Winter  Youth Olympic Games 2012 in Innsbruck, Austria | Not systematic review |
| Fridman et al., 2013 | Epidemiology of Sports-related Injuries in Children  and Youth Presenting to Canadian Emergency  Departments from 2007–2010 | Not systematic review |
| Pocecco et al., 2013 | Injuries in Judo - A Systematic Literature Review Including Suggestions for Prevention | Design |
| Richmond et al., 2013 | Is Body Mass Index a Risk Factor for Sport Injury in Adolescents? | Not systematic review |
| Stracciolini et al., 2013 | Pediatric Sports Injuries. An Age Comparison of Children Versus Adolescents | Not systematic review |
| Abrahams et al., 2014 | Risk Factors for Sports Concussion: An Evidence-based Systematic Review | Not topic |
| LaBella et al., 2014 | Anterior Cruciate Ligament Injuries: Diagnosis, Treatment, and Prevention | Not systematic review |
| Rössler et al., 2014 | Exercise-based Injury Prevention in Child and Adolescent Sport | Not topic |
| Nalliah et al., 2014 | Epidemiology of Hospital-Based Emergency Department Visits Due to Sports Injuries | Not systematic review |
| Nauta et al., 2014 | A Systematic Review on the Effectiveness of School and Community-based Injury Prevention programmes | Not topic |
| Robinson et al., 2014 | Shoulder Injuries Among US High School Athletes, 2005/2006–2011/2012 | Not systematic review |
| Rössler et al., 2014 | Exercise-Based Injury Prevention in Child and Adolescent Sport: A Systematic Review and Meta-Analysis | No relevant data |
| Stege et al., 2014 | Effect of Interventions on Potential, Modifiable Risk Factors for Knee Injury in Team Ball Sports: A Systematic Review | Not topic |
| Theisen et al., 2014 | Injuries in Youth Sports: Epidemiology, Risk Factors and Prevention | Not systematic review |
| Dobnik, 2015 | Injury Risk Factors in Children and Youth in Physical/Sports Activity | Not systematic review |
| Grantham et al., 2015 | The Curveball as a Risk Factor for Injury: A Systematic Review | Not topic |
| Leppänen et al., 2015 | Overuse Injuries in Youth Basketball and Floorball | Not systematic review |
| Soomro et al., 2015 | The Efficacy of Injury Prevention Programs in Adolescent Team Sports. A Meta-analysis | Not topic |
| Roos et al., 2015 | Epidemiology of Overuse Injuries in Collegiate and High School Athletics in the US | Not systematic review |
| Schroeder et al., 2015 | Epidemiology of Overuse Injuries among High-School Athletes in the United States | Not systematic review |
| Whittaker et al., 2015 | Risk Factors for Groin Injury in Sport: An Updated Systematic Review | Not age group |
| Baugh et al., 2016 | High School Rowing Injuries: National Athletic Treatment, Injury and Outcomes Network (NATION) | Not systematic review |
| McKenzie et al., 2016 | Epidemiology of Skateboarding-related Injuries Sustained by Children and Adolescents 5-19 years of Age and Treated in US Emergency Departments: 1990  through 2008 | Not systematic review |
| O' Connor et al., 2016 | Epidemilogy of Injury in Male Adolescent Gaelic Games | Not systematic review |
| Pfister et al., 2016 | The Incidence of Concussion in Youth Sports:  A Systematic Review and Meta-analysis | No relevant data |
| Embree et al., 2016 | Risk Factors for Bicycling Injuries in Children and Adolescents: A Systematic Review | Not topic |
| Fabricant et al., 2016 | Youth Sports Specialization and Musculoskeletal Injury: A Systematic Review of the Literature | Design |
| Stracciolini et al., 2017 | Injury Prevention in Youth Sports | Not systematic review |
| Walters et al., 2017 | The Effects of Resistance Training, Overtraining, and Early Specialization on Youth Athlete injury and Development: A Literature Review | Not topic |
| Burchard et al., 2017 | School Sport-associated Injuries in  Adolescents: A Single Center Experience | Not systematic review |
| Čierna and Lystad, 2017 | Epidemiology of Competition Injuries in Youth Karate  Athletes: A Prospective Cohort Study | Not systematic review |
| Fuglkjær et al., 2017 | Prevalence and Incidence of Musculoskeletal Extremity Complaints in Children and Adolescents. A systematic review | Not topic |
| Hashemi et al., 2017 | A Systematic Review on the Epidemiology of Pediatric Burn in Iran | Not topic |
| Zwolski et al., 2017 | Resistance Training in Youth: Laying the Foundation for Injury Prevention and Physical Literacy | Not topic |
| Pasulka et al., 2017 | Specialization Patterns across Various Youth Sports and Relationship to Injury Risk | Not systematic review |
| Makdissi et al., 2017 | Approach to Investigation and Treatment of Persistent Symptoms Following Sport-related Concussion | Not topic |
| Patel et al., 2017 | Epidemiology of Sports-related Musculoskeletal Injuries in Young Athletes in United States | Not systematic review |
| Ukogu et al., 2017 | Epidemiology of Youth Sports Injury: A Review of Demographic and Sports-related Risk Factors for Injury | Not systematic review |
| Asker et al., 2018 | Risk Factors for, and Prevention of, Shoulder Injuries in Overhead Sports: A Systematic Review with Best-evidence Synthesis | Not topic |
| Bao et al., 2018 | Lack of Sleep and Sports Injuries in Adolescents: A Systematiac Review and Meta-analysis | Not topic |
| Bell et al., 2018 | Sport Specialization and Risk of Overuse Injuries: A Systematic Review With Meta-analysis | Not topic |
| Calderazzi et al., 2018 | Apophyseal Avulsion Fractures of the Pelvis. A review | Not topic |
| Halstead et al., 2018 | Sport-Related Concussion in Children and Adolescents | Not systematic review |
| Han et al., 2018 | Epidemiology of Figure Skating Injuries: A Review of Literature | Not systematic review |
| Knapik et al., 2018 | Prevalence and Management of Coracoid Fracture Sustained During Sporting Activities and Time to Return to Sport. A Systematic Review | Not topic |
| Li et al., 2018 | Child injuries in Ethiopia: A Review of the  Current Situation with Projections | Not systematic review |
| Perera et al., 2018 | Epidemiology of Injuries in Women Playing Competitive Team Bat-or-Stick Sports: A Systematic Review and a Meta-Analysis | Not age group |
| Richmond et al., 2018 | A Systematic Review of the Risk Factors and Interventions for the Prevention of Playground Injuries | Not topic |
| Storm et al., 2018 | The Relationship Between Range of Motion and Injuries in Adolescent Dancers and Sportspersons: A Systematic Review | Not topic |
| Zonfrillo et al., 2018 | A Systematic Review of Longitudinal Cohort Studies Examining Unintentional Injury in Young Children | Not age group |
| Bergeron et al., 2019 | Don’t Take Down the Monkey Bars. Rapid Systematic Review of Playground-related Injuries | No relevant data |
| Borel et al., 2019 | Prevalence of Injuries in Brazilian Recretional Street Runners: Meta-analysis | Not age group |
| Brown and Moran, 2019 | Pediatric Sports-related Injuries | Not systematic review |
| Eapen et al., 2019 | Clinically Important Sport-related Traumatic Brain  Injuries in Children | Not systematic review |
| Norton et al., 2019 | Risk Factors for Elbow and Shoulder Injuries in Adolescent Baseball Players. A Systematic Review | Not topic |
| Scheffler et al., 2019 | Contact Sport Related Head and Neck Injuries in Pediatric Athletes | Not systematic review |
| Burger et al., 2020 | Lay of the Land: Narrative Synthesis of Tackle Research in Rugby Union and Rugby Sevens | Not age group |
| Carder et al., 2020 | The Concept of Sport Sampling Versus Sport Specialization: Preventing Youth Athlete Injury. A Systematic Review and Meta-analysis | Not topic |
| Crossley et al., 2020 | Making Football Safer for Women: A Systematic Review and Meta-analysis of Injury Prevention Programmes in 11 773 Female Football (Soccer) Players | Not topic |
| Deal et al., 2020 | Regional Interdependence and the Role of the Lower Body in Elbow Injury in Baseball Players. A Systematic Review | Not topic |
| Fajardo Pulido and Lystad, 2020 | Epidemiology of Injuries in Ultimate (Frisbee): A Systematic Review | Not age group |
| McLeod et al., 2020 | Supplementary data for article Prospective Reporting of Injury in Community-level Cricket: A Systematic Review to Identify Research Priorities | Not relevant material |
| Muljadi et al., 2020 | Comparative Surgical Risk between Type of Trampoline (size and place) and Type of Patients (age and sex) in Trampoline Related Injury: A Systematic Review and indirect Meta-analysis | Not topic |
| Sollerhed et al., 2020 | Adolescent Physical Activity-related Injuries in School Physical Education and Leisure-time Sports | Not systematic review |
| Sommerfield et al., 2020 | A Prospective Study of Sport Injuries in Youth Females | Not systematic review |
| Werlich et al., 2020 | Prevalence of Dentofacial Injuries in Contact Sports Players: A Systematic Review and Meta-analysis | Not age group |
| Račaitė et al., 2021 | Parent Emigration, Physical Health and Related Risk and Preventive Factors of Children Left Behind: A Systematic Review of Literature | Not topic |
