# Supplementary material for: Frequency of Physical Activity-Related Injuries Among Adolescents: An Umbrella Review
Source: Public Health Rev. 2025 Jan 22;46:1606767. doi: 10.3389/phrs.2025.1606767 (PMC11815350; doi:10.3389/phrs.2025.1606767)
Supplement: Supplementary file 3 [file Table3.DOCX]

Supplementary file 3: List of excluded studies after quality assessment (n = 49)

| **Study** | **Title** | **Reason for exclusion** |
| --- | --- | --- |
| Macera and Wooten, 1994 | Epidemiology of Sports and Recreation Injuries Among Adolescents | Low quality |
| Duggleby and Kumar, 1997 | Epidemiology of Juvenile Low Back Pain: A Review | Low quality |
| Baker and Patel, 2000 | Sports Related Mild Traumatic Brain Injury in Adolescents | Low quality |
| Benson and Meeuwisse, 2005 | Ice Hockey Injuries | Low quality |
| Caine and Nassar, 2005 | Gymnastics injuries | Low quality |
| Giza and Micheli, 2005 | Soccer Injuries | Low quality |
| Hagel, 2005 | Skiing and Snowboarding Injuries | Low quality |
| Harmer, 2005 | Basketball Injuries | Low quality |
| Hewett et al., 2005 | Wrestling Injuries | Low quality |
| Kibler and Safran, 2005 | Tennis Injuries | Low quality |
| Lyman and Fleisig, 2005 | Baseball Injuries | Low quality |
| McCrory and Turner, 2005 | Equestrian Injuries | Low quality |
| McIntosh, 2005 | Rugby Injuries | Low quality |
| Stuart, 2005 | Football Injuries | Low quality |
| Toth et al., 2005 | Central Nervous System Injuries in  Sport and Recreation | Low quality |
| Caine et al., 2006 | Incidence and Distribution of Pediatric Sport-Related Injuries | Low quality |
| Ackery et al., 2007 | An International Review of Head and Spinal Cord Injuries in Alpine Skiing and Snowboarding | Low quality |
| Louw et al., 2007 | Epidemiology of Knee Injuries among Adolescents:  A Systematic Review | Low quality |
| Magra et al., 2007 | A Review of Epidemiology of Paediatric Elbow Injuries in Sports | Low quality |
| Spinks and McClure, 2007 | Quantifying the Risk of Sports Injury | Low quality |
| Caine et al., 2008 | Epidemiology of Injury in Child and Adolescent Sports: Injury Rates, Risk Factors, and Prevention | Low quality |
| Collard et al., 2008 | Acute Physical Activity and Sports Injuries in  Children | Low quality |
| Knowles, 2009 | Is There an Injury Epidemic in Girls’ Sports? | Low quality |
| Colvin and Lynn, 2010 | Sports-Related Injuries in the Young Female Athlete | Low quality |
| Steffen and Engebretsen, 2010 | More Data Needed on Injury Risk among Elite Athletes | Low quality |
| Zemper, 2010 | Catastrophic Injuries Among Young Athletes | Low quality |
| Bleakley et al., 2011 | Epidemiology of Adolescent Rugby Injuries: A Systematic Review | Low quality |
| Shea et al., 2011 | Youth Sports Anterior Cruciate Ligament and Knee Injury Epidemiology: Who Is Getting Injured? In What Sports? When? | Low quality |
| Hunter, 2012 | Hunter The Epidemiology of Injury in Skateboarding | Low quality |
| Larson and McIntosh, 2012 | The Epidemiology of Injury in ATV and Motocross Sports | Low quality |
| Faude et al., 2013 | Football Injuries in Children and Adolescent Players: Are There Clues for Prevention? | Low quality |
| Sobhani et al., 2013 | Epidemiology of Ankle and Foot Overuse Injuries in Sports: A Systematic Review | Low quality |
| Williams et al., 2013 | A Meta-Analysis of Soccer Injuries on Artificial Turf and Natural Grass | Low quality |
| Nauta et al., 2014 | Injury Risk During Different Physical Activity Behaviours in Children: A Systematic Review with Bias Assessment | Low quality |
| American Academy of Pediatrics, 2015 | Tackling in Youth Football | Low quality |
| Azami-Agdash et al., 2015 | Prevalence, Etiology, and Types of Dental Trauma | Low quality |
| Shi et al., 2015 | Unintentional Injuries in Children with  Disabilities: A Systematic Review and Meta-analysis | Low quality |
| Longo et al., 2016 | Apophyseal Injuries in Children’s and Youth Sports | Low quality |
| Onate et al., 2016 | Physical Exam Risk Factors for Lower Extremity Injury in  High School Athletes: A Systematic Review | Low quality |
| Pfirrmann et al., 2016 | Analysis of Injury Incidences in Male Professional Adult and Elite Youth Soccer Players: A Systematic  Review | Low quality |
| Wiggins et al., 2016 | Risk of Secondary Injury in Younger Athletes After Anterior Cruciate Ligament Reconstruction | Low quality |
| Trentacosta et al., 2018 | Hip and Groin Injuries in Dancers: A Systematic Review | Low quality |
| Andreoli et al., 2018 | Epidemiology of Sports Injuries in Basketball: Integrative Systematic Review | Low quality |
| Caine et al., 2018 | Pediatric and Adolescent Injury in Mountain Biking | Low quality |
| Anderson et al., 2019 | Epidemiology of Injuries in Ice Hockey | Low quality |
| Leahy et al., 2019 | Injury Surveillance in School Rugby: A Systematic Review of Injury Epidemiology & Surveillance Practices | Low quality |
| Watson and Mjaanes 2019 | Soccer Injuries in Children and Adolescents | Low quality |
| Anderson et al. 2020 | Lower Limb MSK Injuries among School-aged Rugby and Football Players: A Systematic Review | Low quality |
| Prakash, 2020 | Epidemiology of High Ankle Sprains. A Systematic Review | Low quality |
